# Estrogenic Activity of Bisphenol A and 2,2-bis(*p*-Hydroxyphenyl)-1,1,1-trichloroethane (HPTE) Demonstrated in Mouse Uterine Gene Profiles

**DOI:** 10.1289/ehp.1002347

**Published:** 2010-09-08

**Authors:** Sylvia C. Hewitt, Kenneth S. Korach

**Affiliations:** Receptor Biology Section, Laboratory of Reproductive and Developmental Toxicology, National Institute of Environmental Health Sciences, National Institutes of Health, Department of Health and Human Services, Research Triangle Park, North Carolina, USA

**Keywords:** BPA, ERα, estrogen, HPTE, microarray, uterus

## Abstract

**Background:**

Interest and concern regarding potentially estrogenic substances have resulted in development of model systems to evaluate mechanisms of such chemicals. Microarray studies have indicated that estradiol (E_2_)-stimulated uterine responses can be divided into early and late phases. Comparison of E_2_ uterine transcript profiles and those of other estrogenic chemicals of interest *in vivo* indicates mechanisms and activities of test compounds.

**Objectives:**

We compared transcript responses and mechanisms of response using mouse reproductive tracts after treatment with E_2_, estriol (E_3_), bisphenol A (BPA), and 2,2-bis(*p*-hydroxyphenyl)-1,1,1-trichloroethane (HPTE).

**Methods:**

Uterine RNA from ovariectomized wild-type mice, estrogen receptor α (ERα) knockout (αERKO) mice, and mice expressing a DNA-binding–deficient ERα (KIKO) treated with E_2_, E_3_, BPA, or HPTE for 2 or 24 hr was analyzed by microarray. Resulting regulated transcripts were compared by hierarchical clustering and correlation analysis, and response patterns were verified by reverse-transcription real-time polymerase chain reaction (RT-PCR).

**Results:**

Both xenoestrogens, BPA and HPTE, showed profiles highly correlated to that of E_2_ in the early response phase (2 hr), but the correlation diminished in the later response phase (24 hr), similar to the known weak estrogen E_3_. Both xenoestrogens also mimicked E_2_ in samples from KIKO mice, indicating that they are able to utilize the indirect tethering mode of ERα signaling. No response was detected in ERα-null uteri, indicating that ERα mediates the responses.

**Conclusion:**

Our study forms a basis on which patterns of response and molecular mechanisms of potentially estrogenic chemicals can be assessed.

Numerous xenoestrogenic substances interact with estrogen receptor α (ERα) to initiate ERα-mediated events, including stimulation of cell responses such as proliferation and modulation of ERα-dependent gene regulation (Moggs 2005). ERα can modulate gene expression by interacting directly with estrogen-responsive enhancer (ERE) DNA sequences in target genes and then recruiting necessary coregulatory factors to alter transcription rates. Alternatively, ERα can affect the rate of transcription via interaction with other DNA-binding transcription factors, such as AP-1 (activator protein 1) or Sp1 (specificity protein-1), which then interact with their respective DNA motifs, leading to estrogen-dependent control of target genes lacking EREs (Björnström and Sjöberg 2005; O’Lone et al. 2004). Because this second mechanism results in ERα being indirectly “tethered” to DNA motifs, it is sometimes referred to as the tethered mechanism. Studies have indicated that this tethered pathway is sensitive to xenoestrogenic endocrine-disrupting compounds (Fujimoto et al. 2004; Safe and Kim 2008; Wu et al. 2008).

Awareness of the potential for negative impact of exposures to chemicals has led investigators to focus on possible endocrine-disrupting xenoestrogenic chemicals, which disrupt or interfere with normal endocrine signals (Dey et al. 2009; Diamanti-Kandarakis et al. 2009; Gray et al. 2009; Miller et al. 2006; Moggs 2005). One such chemical that can impact ERα responses is bisphenol A (BPA). BPA was initially developed as a synthetic estrogen (Dodds et al. 1937) but subsequently has been used to manufacture polycarbonate, a plastic polymer. BPA elicits ERα-dependent uterine weight increase (Markey et al. 2001), epithelial proliferation, and the up-regulation of insulin-like growth factor 1 (*Igf-1*) transcript (Klotz et al. 2000). Recent concern has developed regarding the potential negative impacts of BPA because of its widespread use in polycarbonate plastics in food containers.

2,2-bis(*p*-Hydroxyphenyl)-1,1,1-trichloroethane (HPTE), an estrogenic metabolite of the pesticide methoxychlor, behaves similarly to estradiol (E_2_) and BPA in uterine response studies (Klotz et al. 2000; Newbold et al. 2001). Much data has accumulated indicating that HPTE has biological estrogenic activities; however, open questions remain regarding the molecular players underlying the biological events. Methoxychlor has proven toxicity that affects ovarian functions (Borgeest et al. 2002; Miller et al. 2006) and appears to cause these effects because of interactions with ERα, ERβ, and the androgen receptor (AR) (Gaido et al. 2000; Waters et al. 2001). More specifically, from *in vitro* cell studies, HPTE appears to exhibit agonist activity with ERα and antagonist activity with ERβ or AR (Gaido et al. 1999, 2000).

We have developed microarray profiling of the mouse uterus as a sensitive and comprehensive approach to study the direct targets of E_2_ and xenoestrogens (Hewitt et al. 2003). By evaluating uterine transcript profiles of estrogenic substances such as BPA and HPTE with our model system, we hope to understand the extent to which such compounds can initiate ERα-mediated gene regulation or mediate other non-ERα responses. As part of our investigations into mechanisms of estrogen response in the mouse uterine model, we have used an ERα knock-in mouse that carries a mutation in its ERα, rendering it unable to directly bind to DNA, thus restricting it to the tethered mode of ERα-mediated gene responses. Because female mice carrying one copy of the ERα knock in mutation are infertile, the line has been intercrossed with the ERα knockout (αERKO) line to generate females with one knock-in (KI) and one knockout (KO) *ER*α allele (O’Brien et al. 2006). These mice are thus referred to as ERα KIKO. Our previous studies indicated that ERα KIKO mice selectively retain some uterine gene responses to E_2_ and also exhibit transcriptional responses unique to KIKO mice (Hewitt et al. 2009). Xenoestrogens also employ the tethered mechanism; therefore, gene profiles for BPA and HPTE in uteri of KIKO mice were evaluated and compared with profiles of uteri from wild-type (WT) mice to determine whether these compounds are active via this mechanism *in vivo* and to see if BPA or HPTE exhibits any unique responses.

## Materials and Methods

### Animals

All animal studies were in accordance with National Institutes of Health guidelines (Institute of Laboratory Animal Resources 1996) and an animal studies protocol approved by the National Institute of Environmental Health Sciences (NIEHS) Animal Care and Use Committee. The animals were treated humanely and with regard for alleviation of suffering.

Animals were either an ERα-null line (αERKO) (Lubahn et al. 1993), maintained at Taconic Farms (Germantown, NY), or were obtained by crossing the tethered-selective nonclassical ER knock-in (NERKI) heterozygous males (WT/KI) with αERKO heterozygous females (WT/KO). These crosses were done at Charles River (Wilmington, MA). Offspring were screened for the presence of the *ER*α*KO* and/or *ER*α*KI* alleles as previously described (Hewitt et al. 2009). Females that carried one copy each of the *ER*α*KI* and *ER*α*KO* alleles (KIKO), as well as ERα WT mice, were shipped to the NIEHS. Homozygous αERKO females were shipped from Taconic Farms. All mice were ovariectomized after reaching at least 10 weeks of age, rested for 10–14 days, and then used in studies.

### Microarray

Ovariectomized WT, KIKO, and αERKO mice were injected intraperitoneally with 100 μL 0.85% saline/0.25% ethanol (EtOH) vehicle or with 2.5 μg/mL E_2_ or estriol (E_3_) in 0.85% saline/0.25% EtOH (10 μg/kg body weight) for the E_2_ and E_3_ profiles. For the BPA and HPTE profiles, mice were injected subcutaneously with 100 μL 4% EtOH/sesame oil (Sigma Chemical Co., St. Louis, MO) vehicle or 100 μL 7.5 mg/mL BPA (Sigma) or HPTE (kindly provided by W.N. Jefferson, NIEHS) dissolved in 4% EtOH/sesame oil. Both were used at a dose (750 μg/mouse or 30 mg/kg) that was previously selected to initiate IGF1 receptor–mediated signaling as well as ERα-dependent uterine epithelial proliferation to a level comparable to that of E_2_ (Klotz et al. 2000). Tissue was collected after 2 or 24 hr of oil or saline vehicle, E_2_, E_3_, BPA, or HPTE injection because these time points have previously demonstrated a representative sampling “snapshot” of ERα-dependent uterine gene responses at the two major phases of uterine actions (Hewitt et al. 2003). Uterine tissue (three to five uteri per group) was collected and snap-frozen in liquid nitrogen, and RNA was prepared from the pooled tissue and analyzed as previously described (Hewitt et al. 2003).

For E_2_, BPA, and HPTE, we conducted gene expression analyses using Agilent Mouse Oligo arrays (pattern 011978; Agilent Technologies, Palo Alto, CA) using two-color hybridization. Total RNA was amplified using the Agilent Low RNA Input Fluorescent Linear Amplification Kit protocol. Cy3 or Cy5-labeled cRNA was produced according to the manufacturer’s protocol from 500 ng total RNA per sample. For each two-color comparison, 750 ng each of Cy3 and Cy5-labeled cRNAs (oil or saline vehicle control and E_2_-, BPA-, or HPTE-treated from the same genotype) were mixed and fragmented using the Agilent In Situ Hybridization Kit (Agilent Technologies) following the manufacturer’s protocol. Hybridizations were performed for 17 hr in a rotating hybridization oven using the Agilent 60-mer oligo microarray processing protocol. Two slides were hybridized for each sample pairing to allow for dye reversals (technical replicates). Slides were washed as indicated in the protocol and then scanned with an Agilent Scanner. Data were obtained using Agilent Feature Extraction software (version 7.5), using defaults for all parameters. This software performed error modeling, adjusting for additive and multiplicative noise. The resulting data were processed using the Rosetta Resolver system (version 7.2; Rosetta Biosoftware, Kirkland, WA). The ratio intensity value for each gene feature on the array was averaged across technical replicates using the error-weighted approach described by Weng et al. (2006). A *p*-value for each gene probe was computed based on the reproducibility of the expression measurements across the technical replicates. Gene features with *p* < 0.001 were considered differentially expressed. Data were also filtered to exclude probes for which the signal intensity was not > 100 in any treatment. The resulting lists were combined and clustered hierarchically using Rosetta Resolver.

For E_3_ gene expression, analysis was also conducted using Agilent Whole Mouse Genome 4 × 44 multiplex format oligo arrays (no. 014850; Agilent Technologies) following the manufacturer’s protocol for one-color microarray-based gene expression analysis. Starting with 500 ng total RNA, Cy3-labeled cRNA was produced according to the manufacturer’s protocol. For each sample, 1.65 μg Cy3-labeled cRNA was fragmented and hybridized for 17 hr in a rotating hybridization oven. Slides were washed and then scanned with an Agilent Scanner. Data were obtained using Agilent Feature Extraction software (version 9.5), using the one-color defaults for all parameters. This software performed error modeling, adjusting for additive and multiplicative noise. The resulting data were processed using the Rosetta Resolver system. All data have been deposited in the Gene Expression Omnibus (GEO; accession numbers GSE18168, GSE23241, and GSE24525; National Center for Biotechnology Information 2010).

### Real-time polymerase chain reaction (PCR) to verify array findings

RNA was prepared from animals treated as described for microarray samples (three mice per treatment group). cDNA was prepared from individual uteri and analyzed by SYBR Green real-time PCR using methods and primers previously described (Hewitt et al. 2009, 2010). Computed values for each transcript were relative to WT saline vehicle. Means and SDs were calculated for the three sample group replicates in each treatment and ERα mouse line, and values were compared by one-way analysis of variance (ANOVA) and Tukey’s comparison.

### Immunohistochemistry

Formalin-fixed uterine pieces were embedded on end in paraffin, and cross sections were cut in 4-μm slices, mounted on Superfrost charged slides (Fisher, Pittsburgh, PA), deparaffinized, and hydrated. Ki67 was detected as previously described (Hewitt et al. 2006). Phosphorylated serine 10 (phospho ser10) histone H3 was detected using a similar method, except blocking buffer contained 1.5% goat serum (Santa Cruz Biotechnology, Santa Cruz, CA), 1% bovine serum albumin, and primary antibody (catalog no. 06-570; Upstate Cell Signaling Solutions, Lake Placid, NY) diluted 1:500 in blocking buffer and was incubated on slides for 1 hr.

### Uterine weight bioassay

Ovariectomized WT mice were injected subcutaneously daily with 100 μL 4% EtOH/sesame oil vehicle or 2.5 μg/mL E_2_ or 2.5 μg/mL E_3_ in sesame oil/0.25% EtOH vehicle, or 7.5 mg/mL BPA or HPTE in 4% EtOH/sesame oil vehicle. At 24 hr after the final injection, uteri were collected and weighed (four animals per group).

## Results

### Biological responses

**Figure 1:**
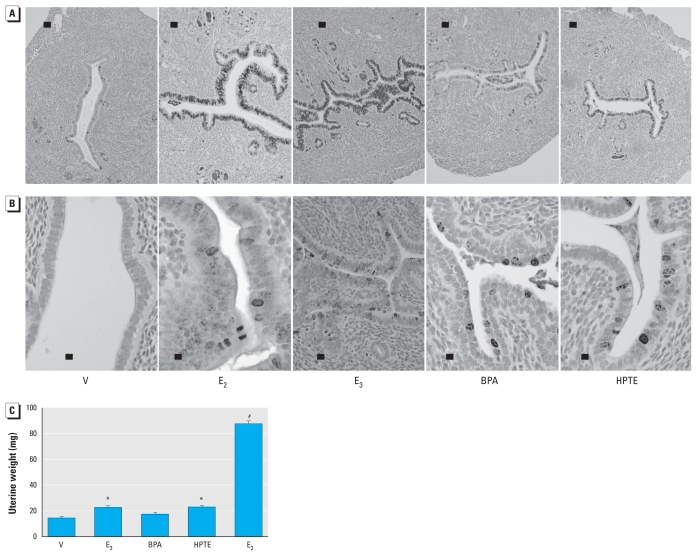
E_2_-, E_3_-, BPA-, and HPTE-mediated uterine growth responses. Photomicrographs showing Ki67 (*A*; bar = 100 μm) and phospho ser10 histone H3 (*B*; bar = 20 μm) in uterine tissue from mice treated 24 hr with oil vehicle (V), E_2_, E_3_, BPA, or HPTE. (*C*) Uterine weight (mean ± SD) of mice injected daily for 3 days with vehicle, E_2_, E_3_, BPA, or HPTE; uterine weights were collected on the fourth day. *p < 0.05, and ^#^*p* < 0.001, compared with vehicle, by one-way ANOVA and Tukey’s comparison; all treatment groups were significantly different (*p* < 0.001) from E_2_.

We evaluated the biological response of the uterine tissues after 24 hr of treatment by detection of the proliferation marker Ki67 (Figure 1A). E_2_ is known to increase Ki67 in the uterine epithelia. The weak estrogen E_3_ (Clark and Markaverich 1984; Katzenellenbogen 1984; Lan and Katzenellenbogen 1976) also increased the marker. Both xenoestrogens, BPA and HPTE, led to increased Ki67 staining in the epithelial cells, reflecting a proliferative response, but the response is blunted compared with that of E_2_ or E_3_. E_2_, E_3_, BPA, or HPTE did not increase Ki67 in KIKO epithelial cells, indicating a lack of proliferative stimulation (data not shown) and emphasizing a requirement for the DNA-binding function of ERα for uterine growth. Further evaluation of WT tissue indicated mitosis by staining for phospho ser10 histone H3, a marker seen in perimitotic cells (Figure 1B). E_2_, E_3_, HPTE, and BPA resulted in increased detection of the marker in uterine epithelial cells, indicating that mitotic progression is stimulated by all three substances. Interestingly, when administered every 24 hr for 3 days, all compounds except BPA elicited a significant increase in uterine weight on the fourth day (Figure 1C), but the increase from HPTE or E_3_ was significantly less than that from E_2_.

### Microarray analysis

**Figure 2:**
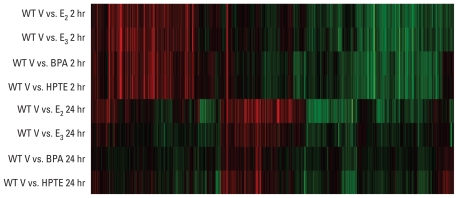
Microarray profile showing comparison of uterine gene response profiles 2 or 24 hr after treatment. Hierarchical clusters were built in Rosetta Resolver using cutoffs of *p* < 0.001 and at least a 2-fold change in expression in WT uteri after E_2_, E_3_, BPA, or HPTE treatment. Each horizontal row represents comparison of saline vehicle (V) and an estrogenic substance (E_2_, E_3_, BPA, or HPTE). Red and green indicate genes that were increased or decreased, respectively, relative to vehicle treatment.

Uterine gene profiles after administration of E_2_, E_3_, or the xenoestrogens BPA or HPTE to ovariectomized WT mice are shown as a heat map representing ratios of transcripts (ratio of treated to vehicle) that are significantly increased (red) or decreased (green) relative to the oil or saline vehicle controls after 2 or 24 hr (Figure 2). The 2-hr responses to E_2_, E_3_, BPA, and HPTE appeared to be very similar. Therefore, scatterplots were generated using Rosetta Resolver software to compare regulated transcripts in vehicle versus BPA or vehicle versus HPTE with vehicle versus E_2_. We observed a highly significant correlation between the compounds [see Supplemental Material, Figure 1 (doi:10.1289/ehp.1002347); correlation coefficients of common signature genes are summarized in Table 1].

Comparisons of uterine transcript responses of tethered-selective ERα KIKO mice and WT mice to E_2_, BPA, or HPTE by microarray [see Supplemental Material, Figure 2 (doi:10.1289/ehp.1002347)] indicate that the KIKO response to all three compounds is very similar. As previously reported for E_2_ (Hewitt et al. 2009), KIKO responses to all three compounds at 2 hr lack some of those seen in WT mice, indicating a requirement for DNA binding for those transcripts (see Supplemental Material, Figure 2A). Also as previously described for E_2_, transcripts unique to the KIKO profiles are apparent with all the compounds. Most responses are absent in the ERα-null αERKO profile at 2 hr (see Supplemental Material, Figure 2A), indicating that the effects of the three compounds are mediated by ERα (WT) or the DNA-binding mutant ERα (KIKO).

Comparisons of the E_2_-, BPA-, and HPTE-regulated KIKO transcripts using scatterplots of the vehicle versus E_2_, compared with vehicle versus BPA or vehicle versus HPTE resulted in correlation coefficients of common signature genes [summarized in Table 2; see also Supplemental Material, Figure 3 (doi:10.1289/ehp.1002347)]. Much like the WT responses, the responses of KIKO uteri to E_2_ and xenoestrogens at 2 hr were highly correlated.

Unlike at the 2-hr time point, the WT responses to E_3_, BPA, and HPTE at 24 hr are less robust than responses to E_2_. The disparity between E_2_ and BPA or HPTE after 24 hr is apparent in the significantly decreased correlation coefficients obtained from a scatterplot of signature genes [Table 1; see also Supplemental Material, Figure 2 (doi:10.1289/ehp.1002347)]. The gene profile seen after treating KIKO mice with E_2_, BPA, or HPTE for 24 hr indicates a weak response, with E_2_ showing the most apparent gene changes, whereas BPA and HPTE show little response [Table 2; see also Supplemental Material, Figure 2B).

The differences in response to E_2_ between WT and KIKO mice noted in an earlier study (Hewitt et al. 2009) are also reflected here in the BPA and HPTE profiles [see Supplemental Material, Figure 2 (doi:10.1289/ehp.1002347)]. Using a scatterplot of WT vehicle versus E_2_ and KIKO vehicle versus E_2_ (Table 2; see also Supplemental Material, Figure 4) to compare these profiles indicates that responses to E_2_ compared with BPA or HPTE within an ERα genotype showed more correlation than when compared with WT and KIKO responses to E_2_. This emphasizes the estrogen-like mechanisms of BPA and HPTE.

### Reverse-transcription real-time (RT)-PCR verification of microarray observations

Overall, the microarray profiles indicate that the xenoestrogens, like the weak estrogen E_3_, were similar to E_2_ in eliciting early gene regulation but less effective in sustaining later responses. This observed trend was verified using RT-PCR evaluation transcripts identified in our previous studies as characteristic of early and late responses to E_2_ (Hewitt et al. 2003, 2005, 2006, 2009).

**Figure 3:**
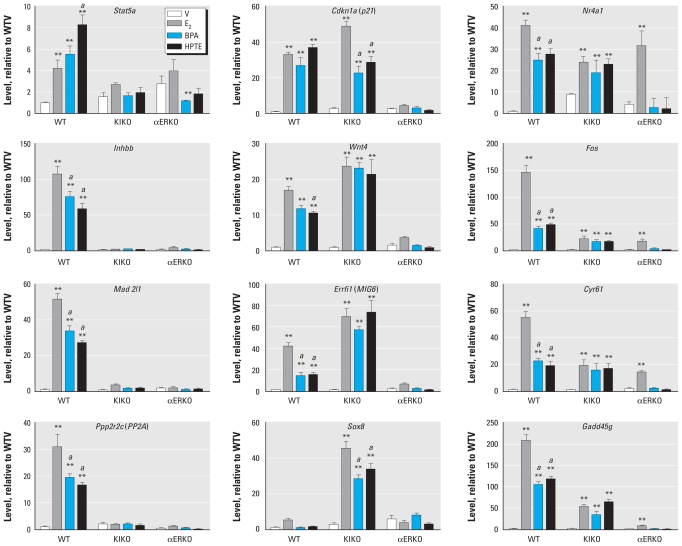
RT-PCR of cDNA prepared from uterine RNA isolated from WT, KIKO, or αERKO mice treated with saline vehicle (V), E_2_, BPA, or HPTE for 2 hr. Abbreviations: *Stat5a,* signal transducer and activator of transcription 5A; *Cdkn1a* (*p21*), cyclin-dependent kinase inhibitor 1A (P21); *Nr4a1*, nuclear receptor subfamily 4, group A, member 1; *Inhbb,* inhibin βb; *Wnt4,* wingless-related MMTV integration site 4; *Fos,* FBJ osteosarcoma oncogene; *Mad2l1*, MAD2 mitotic arrest deficient-like 2 (yeast); *Errfi1* (*MIG6*), ERBB receptor feedback inhibitor 1; *Cyr61*, cysteine rich protein 61; *Ppp2r2c* (*PP2A*), protein phosphatase 2 (formerly 2A), regulatory subunit B (PR 52), gamma isoform; *Sox8,* SRY-box containing gene 8; *Gadd45g,* growth arrest and DNA-damage-inducible 45. Values are calibrated relative to ribosomal protein L7 (PL-7) and are plotted relative to WT vehicle levels for each transcript; results were analyzed by two-way ANOVA with a post *t*-test. *^a^p* < 0.01 relative to E_2_. **p < 0.01 relative to the vehicle within the genotype.

*Stat5a*, *Inhbb*, *Mad2l1*, and *Ppp2r2c* are all transcripts we have previously demonstrated to be increased in the uterus 2 hr after E_2_ treatment in WT but not in KIKO or αERKO mice (Hewitt et al. 2009). All four of these transcripts were increased by E_2_ as well as by BPA and HPTE after 2 hr (Figure 3). None of the compounds could induce these four transcripts in KIKO or αERKO uteri, indicating that the responses rely on full activity of ERα. The xenoestrogens were less effective than E_2_ in inducing *Inhbb*, *Mad2l1*, and *Ppp2r2c*, but HPTE was more effective than E_2_ in increasing *Stat5a*. We previously demonstrated the presence of an ERE in the *Stat5a* promoter, thus explaining the lack of response of this transcript to estrogens in KIKO mice (Hewitt et al. 2010b).

Previous analyses have demonstrated that *Cdkn1a* and *Wnt4* transcripts can be increased by E_2_ in WT and KIKO uteri, indicating that their regulation involves the tethered mechanism (Hewitt et al. 2009; O’Brien et al. 2006). BPA and HPTE also increased *Cdkn1a* and *Wnt4* transcripts in uteri of WT and KIKO mice but not in those of αERKO mice (Figure 3), indicating that these transcripts are regulated by indirect DNA binding. Sp1 binding sites have been identified in both promoters (Ray et al. 2008; Yoshida et al. 2008). BPA and HPTE were as effective as E_2_ in increasing KIKO *Wnt4* and WT *Cdkn1a*. However, in KIKO uteri, *Cdkn1a* induction by BPA or HPTE was significantly lower than induction by E_2_. Similarly, in WT uteri, the increase of *Wnt4* after HPTE exposure was significantly lower than the the increase induced by E_2_. We have previously demonstrated that uterine *Errfi1* is a target of E_2_ and increases in both WT and KIKO samples (Hewitt et al. 2009). Interestingly, BPA and HPTE also increased this transcript in KIKO and WT uteri; although the xenoestrogen-mediated increase in WT uteri is less robust than with E_2_, the increases with BPA and HPTE are equally as effective as those with E_2_ in KIKO uteri.

We previously showed that *Sox8* (SRY-box containing gene 8) transcripts are selectively increased by E_2_ in KIKO but not WT uteri (Hewitt et al. 2009). In the present study, BPA and HPTE also increased *Sox8* selectively in KIKO uteri (Figure 3). E_2_ increased the KIKO *Sox8* transcript more markedly than did BPA or HPTE.

*Nr4a1*, *Fos*, *Cyr61*, and *Gadd45g* are ERα-dependent, rapidly induced uterine transcripts (Hewitt et al. 2003, 2005, 2006, 2009). BPA and HPTE induced WT uterine *Nr4a1*, *Fos*, *Cyr61*, and *Gadd45g* transcripts (Figure 3), but not as robustly as did E_2_. E_2_ increases *Nr4a1*, *Fos*, and *Cyr61* not only in KIKO but also in αERKO uteri (Hewitt et al. 2009), an effect that is likely mediated by residual ERα in KIKO and αERKO uteri from a splice variant, E1, that lacks the N-terminal AF-1 (activation function-1) domain of the ERα (Couse et al. 1995). Here, we found that BPA and HPTE increase *Nr4a1*, *Fos*, and *Cyr61* transcripts in the KIKO but not in the αERKO samples (Figure 3). Thus, it appears that the residual activity mediated through the E1 splice variant in αERKO uteri after E_2_ treatment is not triggered by BPA or HPTE, suggesting that these compounds require the presence of the N-terminal AF-1 region of the ERα. None of these compounds increased *Nr4a1*, *Fos*, or *Cyr61* in uteri of Ex3αERKO, a complete ERα-null model that lacks the E1 splice variant (Hewitt et al. 2010a) [see Supplemental Material, Figure 5 (doi:10.1289/ehp.1002347)]. The increase in these transcripts was equally effective with E_2_ and the xenoestrogens in this experiment (see Supplemental Material, Figure 5), unlike the previous experiment (Figure 3), in which xenoestrogens were less effective than E_2_. The background strain used in the second experiment of WT littermates of the Ex3αERKO mice was predominantly C57bl/6, whereas the WT littermates of the KIKO mice were a mixture of C57bl/J and 129/SvJ, which might alter the sensitivity to or the metabolism of the compounds. Despite the difference in relative effectiveness, these results still indicate that the N-terminal truncated ERα is insensitive to the xenoestrogens and that the responses depend on ERα.

*Gadd45g* is rapidly and robustly increased in both WT and KIKO uteri by E_2_, BPA, and HPTE (Figure 3), but none of the compounds is effective in αERKO uteri, indicating that the response requires ERα. The increase is greater with E_2_ in WT mice.

**Figure 4:**
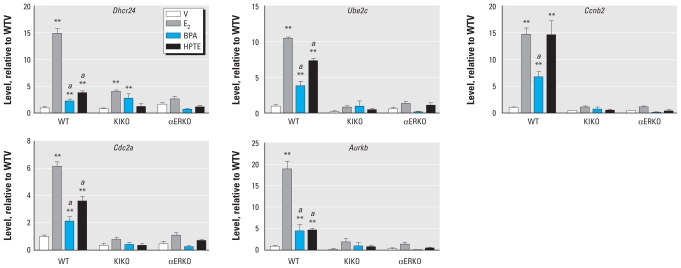
RT-PCR of cDNA prepared from uterine RNA isolated from WT, KIKO, or αERKO mice treated with saline vehicle (V), E_2_, BPA, or HPTE for 24 hr. Abbreviations: *Dhcr24*, 24-dehydrocholesterol reductase; *Ube2c,* ubiquitin-conjugating enzyme E2C; *Ccnb2* cyclin B2; *Cdc2a,* cell division cycle 2 homolog A (*S. pombe*); *Aurkb,* aurora kinase B. Values are calibrated relative to ribosomal protein L7 (PL-7) and are plotted relative to WT vehicle levels for each transcript; results were analyzed by two-way ANOVA with a post *t*-test. *^a^p* < 0.01 relative to E_2_. **p < 0.01 relative to the vehicle within the genotype.

To validate the observed 24-hr gene responses, we selected transcripts that we have previously shown to be regulated by E_2_ (Hewitt et al. 2003, 2005, 2006). *Ube2c*, *Ccnb2*, *Cdc2a*, and *Aurkb* are all associated with the G2/M phases of the cell cycle, and are also all increased in the WT uterus by E_2_ after 24 hr. E_2_ causes little or no increase in these transcripts in KIKO uteri (Hewitt et al. 2010b), which correlated with the lack of uterine growth response. *Dhcr24*, a critical enzyme in cholesterol biosynthesis, is similarly a marker of uterine gene response to E_2_ after 24 hr. BPA and HPTE also increased WT uterine *Dhcr24*, *Ube2c*, *Ccnb2*, *Cdc2a*, and *Aurkb* (Figure 4). The increased transcription was greater with E_2_ than with the xenoestrogens, with the exception of *Ccnb2*, which was increased equally by E_2_ or HPTE but exhibited reduced response to BPA. E_2_, BPA, or HPTE did not increase *Ube2c*, *Ccnb2*, *Cdc2a*, or *Aurkb* in KIKO uteri. KIKO *Dhcr24* was significantly increased by E_2_ or BPA, but the E_2_ increase was markedly less robust than that observed in WT uteri. Overall, analysis of previously characterized ERα-dependent 24-hr uterine transcripts confirms a trend of less responsiveness at the 24-hr time point with BPA and HPTE compared with E_2_ that is also reflected in the attenuated uterine growth (Figure 1B).

### Potential xenoestrogen-selective transcripts could not be verified

Microarray transcript profiles suggest that some ERα-dependent gene responses might have been initiated by BPA and HPTE that were not observed with E_2_. We evaluated five apparent xenoestrogen–up-regulated transcripts observed in the microarray data by RT-PCR of independent samples [see Supplemental Material, Figure 6 (doi:10.1289/ehp.1002347)]. *Axin1* (axis inhibitor 1), *Per1* [period homolog 1 (*Drosophila*)], and *Gna12* (guanine nucleotide binding protein, alpha 12) were all increased by E_2_ as well as BPA and HPTE, whereas *Mvp* (major vault protein) was increased only by BPA, and *Tnxb* (tenascin XB) was not changed by any of the treatments. Thus, except for *Mvp*, none of these genes reproduced the apparent selective xenoestrogen regulation that we observed in the microarray data that were evaluated by RT-PCR with independent samples.

## Discussion

Numerous studies have assessed potential estrogenicity of BPA and HPTE using models more amenable to toxicological and risk assessment (Myers et al. 2009; Tiemann 2008; Vandenberg et al. 2007, 2009; vom Saal et al. 2007). Additionally, chemical modeling studies have indicated possible modes of interaction between BPA or HPTE with ERα compared with E_2_ and other estrogens (Celik et al. 2008). Other approaches that use *in vitro* models have been useful in indicating modes of ER-mediated response (Gaido et al. 1999, 2000; Safe et al. 2001; Yoon et al. 2000) but do not reflect the global transcriptional events or endogenous gene transcriptional regulation. Previous microarray studies have examined prolonged exposures of uterine tissues (Ashby and Odum 2004; Waters et al. 2001) or used cell culture models (Boehme et al. 2009). Our approach differed from these both in method and in purpose. In our study we used methods optimized to be highly estrogen sensitive, not necessarily to address health effects or to address chemical properties, but to indicate aspects of mechanisms and response of these chemicals directly mediated by ERs. Accordingly, although the BPA and HPTE doses were relatively high, they were previously shown to be the minimum doses effective in initiating uterine responses of IGF1 receptor activation and epithelial cell proliferation (Klotz et al. 2000) to a degree comparable to that of E_2_. Similarly, *in vitro* studies have indicated doses of 75 μM BPA and 25 μM HPTE were needed for a reporter gene response to match that of 10 nM E_2_ (Safe and Kim 2008).

Initially, we used microarray to assess the gene profiles of E_2_ compared with two endocrine disruptors: BPA, which is polymerized to produce polycarbonate plastics, and HPTE, which is a metabolite of the pesticide methoxychlor. Both chemicals have been shown to stimulate uterine proliferation in an ERα-dependent manner (Klotz et al. 2000), which we further confirmed here and extended to include evaluation of Ki67 (a general marker of cell proliferation) and phospho ser10 histone H3 (a marker of perimitosis) in addition to uterine weight increase after 72 hr. Interestingly, both BPA and HPTE were less effective than the established weak estrogen E_3_ in stimulating Ki67, but they were similar to E_3_ in all other indicators of uterine growth response. In agreement with a mode similar to the weak estrogen E_3_, the early (2-hr) gene profiles were very similar between E_3_ and E_2_, BPA, or HPTE and further showed dependence on ERα, indicating that these compounds are interacting with ERα to induce transcriptional responses in the uterine tissue. The gene profiles add to a growing body of data that demonstrate ERα-mediated estrogenic activities in a manner that reflects a massive global transcriptional activity that is very highly correlated to the gene responses mediated by E_2_. The reconfirmation of some of these early phase transcripts by RT-PCR indicates that BPA and HPTE are able to regulate uterine transcripts through a mechanism that requires ERα and can use either direct ERE binding or tethered interaction with target genes. Previous work has demonstrated that there are several E_2_-induced transcripts in the uteri of ERα-null αERKO mice. These responses are mediated by a truncated E1 ERα molecule that lacks the AF-1 region (Couse et al. 1995; Hewitt et al. 2009). In the present study, we observed that these E1-responsive transcripts are not sensitive to BPA or HPTE, a novel finding indicating that these compounds rely on the AF-1 cofactor binding region of the ERα. These are compelling findings, suggesting that exposures to chemicals such as BPA or HPTE have the potential to profoundly affect exposed individuals by interfering with endogenous estrogens or by initiating inappropriate estrogenic effects.

We found that the intensities of later (24 hr) E_3_, BPA, and HPTE responses in the gene profiles were reduced relative to E_2_; this suggests that these compounds are weaker than E_2_ in their ability to sustain a response. Indeed, the gene profiles of BPA and HPTE were very similar to that of E_3_, a known weak or impeded estrogen (Clark and Markaverich 1984; Katzenellenbogen 1984). We verified this observed effect by RT-PCR, showing that the xenoestrogens can induce early transcripts similar to E_2_ and that later responses were attenuated. The decrease in the later responses may result from the rates of metabolism or how well BPA and HPTE maintain interaction with ERα, as is seen with E_3_, where retention of nuclear binding and RNA polymerase II activities are similar early after injection but are not maintained as long as E_2_ responses (Clark and Markaverich 1984).

Some transcript responses in the array analysis were apparently unique to the xenoestrogens. Further analysis of signal intensities of these arrays indicated altered basal signal intensity levels of the vehicle channel of BPA and HPTE for about 5–10% of the transcripts in these clusters, representing these hypothetical xenoestrogen-regulated transcripts (data not shown), which gives the appearance that xenoestrogens are regulating these transcripts compared with the altered baseline. Consistent with this, except for one example (*Mvp*), xenoestrogen-selective responses could not be verified by independent RT-PCR. Additionally, we recently repeated this experiment with new samples and no longer saw these patterns (data not shown; GEO accession no. GSE24525). Our results indicate that BPA and HPTE primarily signal through mechanisms mediated by ERα.

We have previously reported that some of the late response genes are associated with G2–M progression, as would be expected to occur as part of the uterine growth response (Hewitt et al. 2003, 2009). BPA and HPTE are less able to mediate some of these transcripts, including *Cdc2a* and *Aurkb* (Figure 4), whereas BPA is less able to induce *Ccnb2*. The inability of BPA and HPTE to maintain the responses is also reflected in the less intense Ki67 level (Figure 1A) as well as lower maximal uterine weight obtained in 3-day uterine bioassays (Figure 1B) (Markey et al. 2001; Newbold et al. 2001).

Overall, the analysis of KIKO responses [which are restricted to the ERE-independent mode of ERα-dependent response (Jakacka et al. 2001; O’Brien et al. 2006)] to the endocrine-disrupting chemicals (BPA, HPTE) indicated similarity with the KIKO response to E_2_, confirming the sensitivity of this mode of ERα signaling for xenoestrogenic compounds. It is interesting that the pattern of response to E_2_ or xenostrogen is more highly correlated within each genotype (WT or KIKO) than with chemical identity, which emphasizes the similarity of the activity of these chemicals to estrogens and the potential for effects *in vivo*. The differences between WT and KIKO gene profiles were the focus of a previously published study (Hewitt et al. 2009). In that study, analyses of the E_2_ responses in KIKO uteri indicated that some pathways (e.g., JAK/STAT signaling) are regulated similarly in WT and KIKO uteri, whereas others (e.g., WNT/β-catenin signaling) are impacted in both WT and KIKO uteri; however, the outcome is altered in KIKO compared with the WT uteri because different members of the pathway are affected or regulated in opposite directions. In the present study, these differences were also observed with BPA and HPTE. For example, the *Ppp2r2c* transcript, encoding an important activator of β-catenin (CTNNB1) transcriptional activity (Gordon and Nusse 2006), was increased in WT uteri by E_2_, as well as by BPA and HPTE (Figure 3), but was not changed in KIKO mice by any of the treatments. Conversely, *Sox8*, which encodes an inhibitor of CTNNB1-mediated transcription (Gordon and Nusse 2006), was not changed in WT uteri but was increased by all three treatments in KIKO uteri (Figure 3). The observed KIKO-selective transcript regulation, such as the increase in *Sox8*, suggests a complex mechanism of regulation or, alternatively, indicates that the cells of the KIKO uterus have developed abnormal signaling pathways. At this point, our study does not address reasons for the unique and unexpected KIKO uterine transcript profile. However, the fact that BPA and HPTE elicited the same unique KIKO uterine transcript profiles as E_2_ supports the idea that their activity and mechanism resemble those of E_2_. Thus, here we present a novel *in vivo* study indicating that, as previously described using *in vitro* cell culture systems (Fujimoto et al. 2004; Safe and Kim 2008; Wu et al. 2008), the tethered response mechanism is sensitive to xenoestrogen compounds.

## Conclusion

Our study was designed to evaluate the mechanistic aspects of xenoestrogenic chemicals in an *in vivo* system and their impact through ERα signaling on biological processes. We observed clear similarities between the xenoestrogens tested and E_2_—especially in the 2-hr treatment group—in the microarray profiles, and we verified these observations by RT-PCR. Our findings and the availability of the microarray data set will be useful in the development of a panel of biomarker uterine transcripts for future evaluation of modes of action and mechanisms of other potentially estrogenic chemicals.

## Figures and Tables

**Table 1:** Correlation of common signature genes in WT mice.

	Oil vehicle	
Saline vehicle	Versus BPA	Versus HPTE
Versus E_2_ 2 hr	0.92 (3,400)	0.92 (3,143)
Versus E_2_ 24 hr	0.61 (1,113)#	0.66 (2,022)#

Values are from scatterplot analyses shown in Supplemental Material, Figure 1A (doi:10.1289/ehp.1002347). The number of common signature genes is shown in parentheses.

# Correlation coefficient is significantly lower (*p* < 0.001) than at 2 hr.

**Table 2:** Correlation of common signature genes in KIKO mice (vs. KIKO E_2_ or WT E_2_).

KIKO vehicle (saline)	KIKO vehicle (oil)		WT saline vehicle versus E_2_
	Versus BPA	Versus HPTE	
Versus E_2_ 2 hr	0.93 (1,896)	0.93 (2,002)	0.77 (2,443)##
Versus E_2_ 24 hr	0.05 (170)#	0.87 (349)#	0.63 (1,433)##

Values are from scatterplot analyses shown in Supplemental Material, Figure 1B and 1C (doi:10.1289/ehp.1002347). The number of common signature genes is shown in parentheses.

# Correlation coefficient is significantly lower (*p* < 0.001) than at 2 hr.

## Correlation coefficient for WT vehicle versus E_2_ versus KIKO vehicle versus E_2_ is significantly lower (*p* < 0.001) than for KIKO vehicle versus E_2_ versus KIKO vehicle versus BPA or HPTE.
